# Real-time evaluation of antibacterial efficacy using bioluminescent assays for *Pseudomonas aeruginosa* and *Staphylococcus aureus*

**DOI:** 10.3389/fmicb.2025.1569217

**Published:** 2025-08-29

**Authors:** Manali Patil, Congyu Luo, Ganna Petruk, Jitka Petrlova, Artur Schmidtchen, Manoj Puthia

**Affiliations:** ^1^Division of Dermatology and Venereology, Department of Clinical Sciences, Lund University, Lund, Sweden; ^2^Faculty of Health and Society, Department of Biomedical Science, Biofilms Research Centre for Biointerfaces, Malmö University, Malmö, Sweden; ^3^Department of Dermatology, Skåne University Hospital, Lund, Sweden

**Keywords:** bioluminescent assay, antibiotic resistance, luciferase, *Staphylococcus aureus*, *Pseudomonas aeruginosa*, antimicrobial susceptibility testing, real-time monitoring

## Abstract

The emergence of antibiotic resistance necessitates effective strategies for evaluating antimicrobial agents. Bioluminescent bacteria, either naturally occurring or engineered with modified reporter genes like bacterial luciferase, provide real-time assessment of bacterial viability through light emission. We investigated the antibacterial effects of cefotaxime and doxycycline using bioluminescent strains of *S. aureus* and *P. aeruginosa*, combining optical density measurements with bioluminescence monitoring. Treatment with cefotaxime resulted in a significant reduction of the bioluminescent signal in *P. aeruginosa* compared to untreated controls, while doxycycline induced a delayed growth curve. Both antimicrobials demonstrated strong efficacy against *S. aureus*, as evidenced by decreased bioluminescence signals. Results from bioluminescence assays and classical minimum inhibitory concentration and minimum bactericidal concentration methods showed consistent alignment, validating the bioluminescence approach. This study demonstrates that bioluminescence-based methods offer a reliable, real-time alternative to traditional bacterial viability assays for evaluating antimicrobial efficacy.

## 1 Introduction

Testing for antibiotic susceptibility is crucial across various applications, including drug development, epidemiological studies, and treatment outcome prediction (Kaprou et al., 2021; Gajic et al., 2022; van Belkum et al., 2019). The growing challenge of microbial resistance has encouraged the development of novel antimicrobial approaches, including new-generation antibiotics, host defense peptides, and naturally derived molecules (Balouiri et al., 2016; Miethke et al., 2021). This has increased the focus on developing robust methods for screening and determining antimicrobial activity (Hossain, 2024).

Various laboratory methods are employed to evaluate *in vitro* antimicrobial effects and determine effective drug concentrations (Hossain, 2024). Traditional approaches include, among others, disk-diffusion and broth or agar dilution methods (Balouiri et al., 2016; Heatley, 1944). For a comprehensive understanding of inhibitory effects—whether bactericidal or bacteriostatic, and whether time-dependent or concentration-dependent—various methods such as time-kill assays and flow cytometry-based approaches are used (Kaprou et al., 2021; Balouiri et al., 2016). Currently, the minimum inhibitory concentration (MIC) is mainly used to address emergence of microbial resistance, monitor antibiotic resistance frequency, and support surveillance studies (Andrews, 2001; Kowalska-Krochmal and Dudek-Wicher, 2021).

The MIC, considered the gold standard, serves as a critical determinant of bacterial strain’s sensitivity or resistance to antibiotics *in vitro* and provides a benchmark for evaluating other susceptibility testing methods. In practice, diagnostic laboratories use MICs to validate abnormal resistance patterns and to provide definitive results when alternative methods yield uncertain results (Sever et al., 2024; Bayot and Bragg, 2025).

However, studies of antimicrobial activity face challenges due to the lack of standardized quantitative approaches (Kaprou et al., 2021; Gonzalez-Pastor et al., 2023). Variations in experimental methods can significantly impact MIC determination. For example, extended incubation periods might elevate MIC values, while lower bacterial inoculum concentrations may artificially reduce them (Laure et al., 2021; Mouton et al., 2018; Smith and Kirby, 2018). Technical differences between laboratories further complicate the comparisons of results. Importantly, MIC only indicates visible growth inhibition without necessarily implying microbial eradication, whereas MBC determines the lowest antimicrobial concentration required to kill ≥99.9% of the bacterial inoculum. The efficacy of antimicrobials depends on multiple factors beyond MIC, including tissue penetration. An antibiotic with a low MIC may exhibit poor activity if it does not achieve adequate tissue penetration and distribution, whereas an antibiotic with a higher MIC may be more effective if it concentrates at the site of infection (Levison and Levison, 2009).

Classical MIC assays are limited by their dependence on visual inspection of bacterial growth. Moreover, they lack real-time information on antimicrobial effects, as results only available after overnight incubation. While optical density measurements provide real-time data, they cannot distinguish between metabolically active and dormant bacteria (Suominen et al., 2020).

Bioluminescent assay techniques utilize either naturally luminescent bacteria or bacteria engineered with reporter genes such as bacterial luciferase (Robinson et al., 2011; Billard and DuBow, 1998). These bacteria emit bioluminescence with a peak wavelength around 490 nm, regardless of luciferase variations (Brodl et al., 2018). Bacterial luminescence serves as an indicator for toxic substances, with the reduction in light emission being proportional to the toxicity levels (Farkas et al., 2024; Ravindran et al., 2014). This has established bioluminescent bacteria as a valuable tool for toxicity studies and antimicrobial evaluation, offering distinct advantages over traditional methods, including real-time metabolic monitoring, non-invasive measurement, and enhanced sensitivity to sublethal effects. The demand for rapid and reproducible *in vitro* and *in vivo* studies has grown significantly, particularly for evaluating antimicrobial formulations (Puthia et al., 2020; Schmidtchen and Puthia, 2022; Puthia et al., 2023). Bioluminescent molecules, especially firefly luciferase, are extensively used in experiments involving pathogen detection, protein interactions, cell communication studies, and antibiotic efficacy evaluation (Schmidtchen and Puthia, 2022; Syed and Anderson, 2021; Chen et al., 2008).

In this study, we demonstrate usefulness of a bioluminescent assay in assessing antimicrobial drug effects using luminescent *P. aeruginosa* and *S. aureus* strains. We employed *P. aeruginosa* Xen 41, expressing the *Photorhabdus luminescens* luxCDABE operon (Winson et al., 1998; Dusane et al., 2017), and *S. aureus* SAP229, containing plasmid pRP1195 with a modified lux operon (Plaut et al., 2013). *P. aeruginosa* and *S. aureus* were selected for their significance in wounds and chronic infections (Conlon, 2014; Phan et al., 2023; Saadatian-Elahi et al., 2008; Serra et al., 2015; Gjodsbol et al., 2006). We evaluated cefotaxime, a beta-lactam antibiotic which inhibits peptidoglycan synthesis in the bacterial cell through PBPs (penicillin-binding proteins) binding (Carmine et al., 1983), and doxycycline, which exhibits broad-spectrum activity by inhibiting bacterial protein synthesis (Cunha et al., 1982). This study serves as a proof-of-concept to demonstrate the feasibility of using bioluminescent bacterial strains for real-time antimicrobial susceptibility testing, providing a foundation for future research with a broader range of species and antibiotics.

## 2 Materials and methods

### 2.1 Reagents

Cefotaxime and doxycycline hyclate were both obtained from Sigma-Aldrich (Saint Louis, MO 63103, USA).

### 2.2 Microorganisms

*Pseudomonas aeruginosa* Xen41 (PerkinElmer, Akron, OH, USA), a bioluminescent strain derived from PAO1, contains a stable chromosomal copy of the *Photorhabdus luminescens* lux operon (Winson et al., 1998; Dusane et al., 2017). *Staphylococcus aureus* SAP229 was kindly provided by Dr. Roger D. Plaut (Division of Bacterial, Parasitic, and Allergenic Products, FDA, Bethesda, MD, USA). This strain contains plasmid pRP1195, which combines a chloramphenicol resistance gene with a modified lux operon from *Photorhabdus luminescens* (Plaut et al., 2013). These genetic modifications enable their use in both *in vitro* and imaging applications.

### 2.3 Growth studies using optical density

For optimal growth conditions and meaningful comparisons, we closely followed standard MIC assay protocols, incorporating minor modifications as needed to suit the specific requirements of our bacterial strains and experimental design. The growth curve studies were performed using a 96-well plate (SPL Life Science, Gyeonggi, Korea). A single colony of the bioluminescent bacterial strains *P. aeruginosa* Xen 41 and *S. aureus* 229 from THA agar plates containing 10 μg/mL of chloramphenicol were inoculated in 3% TH broth (5 mL) and incubated at 37 °C in a shaking incubator. After 16 h, OD of the bacterial solution was measured. The TH media alone was used as a blank. Then, the culture was centrifuged at 3000 × *g* for 10 min at RT. After centrifugation, the supernatant was discarded, and 5 mL of sterile 10 mM Tris HCl (pH 7.4) was added and centrifuged again at 3000 × g for 5 min. Finally, for the growth study, the pellet was resuspended in 2 × BBL™ Mueller Hinton (MH) II cation-adjusted broth (Becton, Dickinson and Company, Sparks, USA) and final concentration was adjusted to 2 × 10^9^ CFU/mL to prepare the stock solution. For the experiment, 100 μL of bacterial suspension at concentrations of 2 × 10^9^ CFU/mL, 1 × 10^7^ CFU/mL, and 5 × 10^5^ CFU/mL was added to a 96-well plate containing 100 μL of MilliQ water. OD readings were measured at 620 nm using a spectrophotometer (Perkin Elmer; VICTOR^3^, 1,420 Multilabel Counter) at 32 °C, with measurements taken hourly for 48 h.

### 2.4 Bioluminescence kinetics studies

The bioluminescence kinetic studies were performed using a 96-well flat-bottom white immunoplate (SPL Lifesciences, Korea). A single colony of the bioluminescent bacterial strains *P. aeruginosa* Xen 41 and *S. aureus* 229 was taken from THA agar plates and inoculated in 3% TH broth (5 mL) and incubated at 37 °C in a shaking incubator. After 16 h, the OD of the bacterial solution was measured at 620 nm. Then, the culture was centrifuged at 3000 × *g* for 10 min at RT. After centrifugation, the supernatant was discarded, and 5 mL of sterile 10 mM Tris HCl (pH 7.4) was added and centrifuged again at 3000 × *g* for 5 min at RT. Finally, for the bioluminescence kinetics study, the pellet was resuspended in 2 × BBL™ Mueller Hinton (MH) II cation-adjusted broth (Becton, Dickinson and Company, Sparks, USA) and the bacterial concentration was adjusted to 2 × 10^9^ CFU/mL and used as stock solution. For the experiment, a final bacterial concentration of 5 × 10^5^ CFU/mL was prepared. Then, 100 μL of the culture was added to a 96-well plate that contained 100 μL of MilliQ water. The bioluminescence readings of the 96-well plate were taken on a luminometer at 32 °C (Perkin Elmer; VICTOR^3^, 1,420 Multilabel Counter) every 1 h until 48 h. All experiments were conducted at 32 °C to minimize evaporation during extended incubation, as higher temperatures led to significant media loss and variability in preliminary trials.

### 2.5 Bioluminescence kinetics studies with antibiotics

To study the effects of antibiotics, a similar experimental setup to that described above for bioluminescence kinetics studies was used. In the final step, 100 μL of the 5 × 10^5^ CFU/mL bacterial suspension was added to a 96-well plate containing 100 μL of MilliQ water with serially diluted antibiotics (cefotaxime and doxycycline) ranging from 0 μg/mL - 20 μg/mL. As previously mentioned, the bioluminescence readings were taken at 32 °C for 48 h. To ensure proper mixing of antibiotics, plates were incubated under gentle shaking.

### 2.6 Minimum inhibitory concentration (MIC) assay

The antibacterial properties of cefotaxime and doxycycline were evaluated using a 96-well round-bottom polystyrene plate (Corning, Inc., Kennebunk, USA). A single colony of the bioluminescent bacterial strains *P. aeruginosa* Xen 41 and *S. aureus* 229 was inoculated into 3% TH broth (5 mL) and incubated at 37 °C in a shaking incubator. After 16 h, the OD of the bacterial solution was measured at 620 nm. A TH broth tube without bacteria was used as a blank to auto-zero the OD. The culture was centrifuged at 3000 × *g* for 10 min at room temperature (RT). After centrifugation, the supernatant was discarded, and 5 mL of sterile 10 mM Tris HCl, pH 7.4, was added and centrifuged again at 3000 × g for 5 min at RT. The pellet was resuspended in Tris HCl, such that the CFU was 2 × 10^9^ CFU/mL. A 1% bacterial solution was diluted in 2 × BBL™ Mueller Hinton (MH) II cation-adjusted broth (Becton, Dickinson and Company, Sparks, USA) to obtain a final bacterial concentration of 5 × 10^5^ CFU/mL. Depending on the number of samples required for the assay, the volumes of cefotaxime and doxycycline needed from a 1 mg/mL stock solution were calculated to achieve a final concentration of 40 μg/mL. 100 μL of MH broth (without the addition of bacteria) was used as a sterile control, 100 μL of MH broth (with the addition of bacteria) was used as a growth control, and 100 μL of water with serially diluted treatment conditions, ranging from 0 to 20 μg/mL of cefotaxime and doxycycline, was prepared for each well. 100 μL of the bacterial solution was added to all wells except the sterile control. A 96-well round-bottom polystyrene plate was incubated at 37 °C with 5% CO_2_ for 24 h before MIC verification. After 24 h, the plates were visually inspected for pellet formation or the presence of turbidity, and this was recorded on the visual inspection report. The MIC value was defined as the point at which no bacterial growth was observed in the well. It was determined to be the lowest treatment concentration necessary to prevent visible bacterial growth in the wells.

### 2.7 Minimum bactericidal concentration (MBC) assay

MBC for the various treatments was then validated using the 96-well round-bottom polystyrene plate from the MIC assay. This procedure involved mixing the bacterial pellet and solution in the wells with a pipette and plating 10 μL droplets on THA plates using an electronic pipette. The plates were then incubated at 37 °C with 5% CO_2_ overnight (the incubation time was 21–24 h). The MBC was defined as the lowest concentration at which no bacterial colonies were observed on the THA plates.

### 2.8 Statistics

All experiments were performed at least 3 times with results expressed as mean ± standard errors. Statistical analyses were conducted using GraphPad Prism 8.0. Differences between treatment groups and untreated controls were evaluated using two-way ANOVA with Fisher’s LSD test. Statistical significance was set at *p* < 0.05. Treatment effects were assessed across time points and concentrations to account for both temporal and dose-dependent responses.

## 3 Results

### 3.1 Growth patterns by optical density measurements

**Figure 1:**
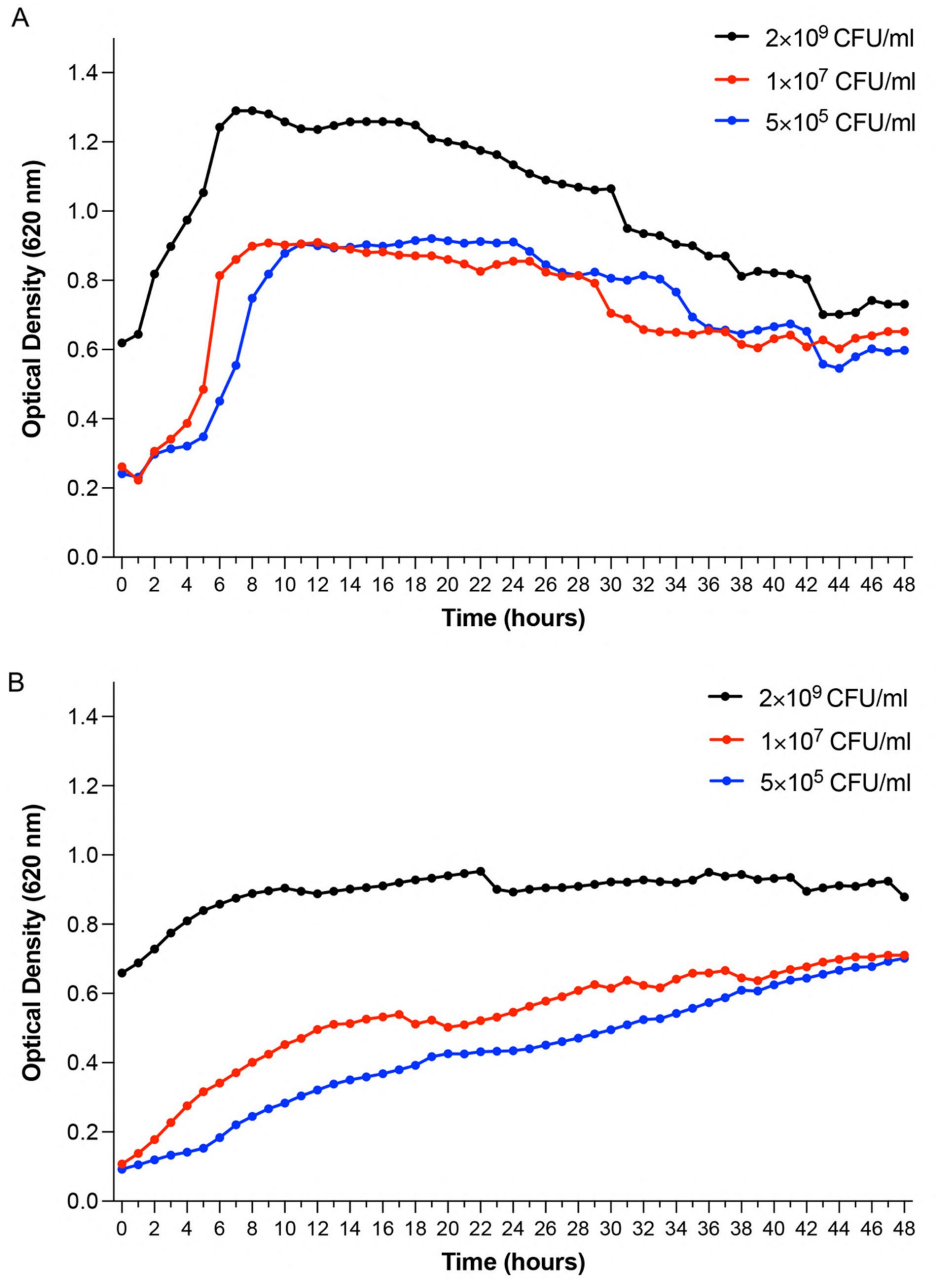
Growth kinetics of *Pseudomonas aeruginosa* Xen 41 and *Staphylococcus aureus* 229 monitored by optical density measurements. Bacterial cultures were started at three different inoculum densities (2 × 10^9^ CFU/mL, 1 × 10^7^ CFU/mL, and 5 × 10^5^ CFU/mL) and monitored continuously for 48 h at 32 °C under aerobic conditions. **(A)** Time-dependent growth profiles of *Pseudomonas aeruginosa* Xen 41 demonstrating concentration-dependent growth patterns and lag-to-exponential phase transitions. **(B)** Time-dependent growth profiles of *Staphylococcus aureus* 229 showing growth characteristics across different initial bacterial densities. Data points represent mean optical density values at 620 nm (OD_620_). Shown is one representative result from three independent experiments (*n = 3*). Growth curves illustrate the relationship between initial inoculum concentration and bacterial growth dynamics over time.

To establish baseline growth characteristics and validate the experimental model, we monitored growth curves of *P. aeruginosa* Xen 41 and *S. aureus* 229 using optical density measurements at 620 nm. These measurements were important to understand normal bacterial growth patterns before evaluating antimicrobial effects. Measurements were conducted in 96-well plates over 48 h at 32 °C. *P. aeruginosa* Xen 41 exhibited characteristic growth phases. Results showed an initial lag phase lasting one hour, followed by exponential growth until hour 9, after which it entered the slow-declining phase (Figure 1A). *S. aureus* 229 showed similar initial lag-phase dynamics but demonstrated sustained growth. After an initial lag phase (~2 h), relatively slower exponential growth commenced, transitioning to stationary phase by 8 h (Figure 1B). These growth studies using 5 × 10^5^, 1 × 10^7^ and 2 × 10^9^ CFU/mL were conducted to account for instrumental limitations in OD measurements and inoculum-dependent growth effects. The 2 × 10^9^ CFU/mL samples exceeded the linear range of the spectrophotometer, which resulted in a distinctly higher OD value, while lower concentrations provided baseline growth dynamics for subsequent antimicrobial assays. While the initial optical density values for 5 × 10^5^ and 1 × 10^7^, CFU/ml appeared similar due to the lower detection threshold of the spectrophotometer, distinct growth curves were observed over time, confirming reliable phase-specific analyses at each inoculum concentration.

### 3.2 Bioluminescence kinetics

**Figure 2:**
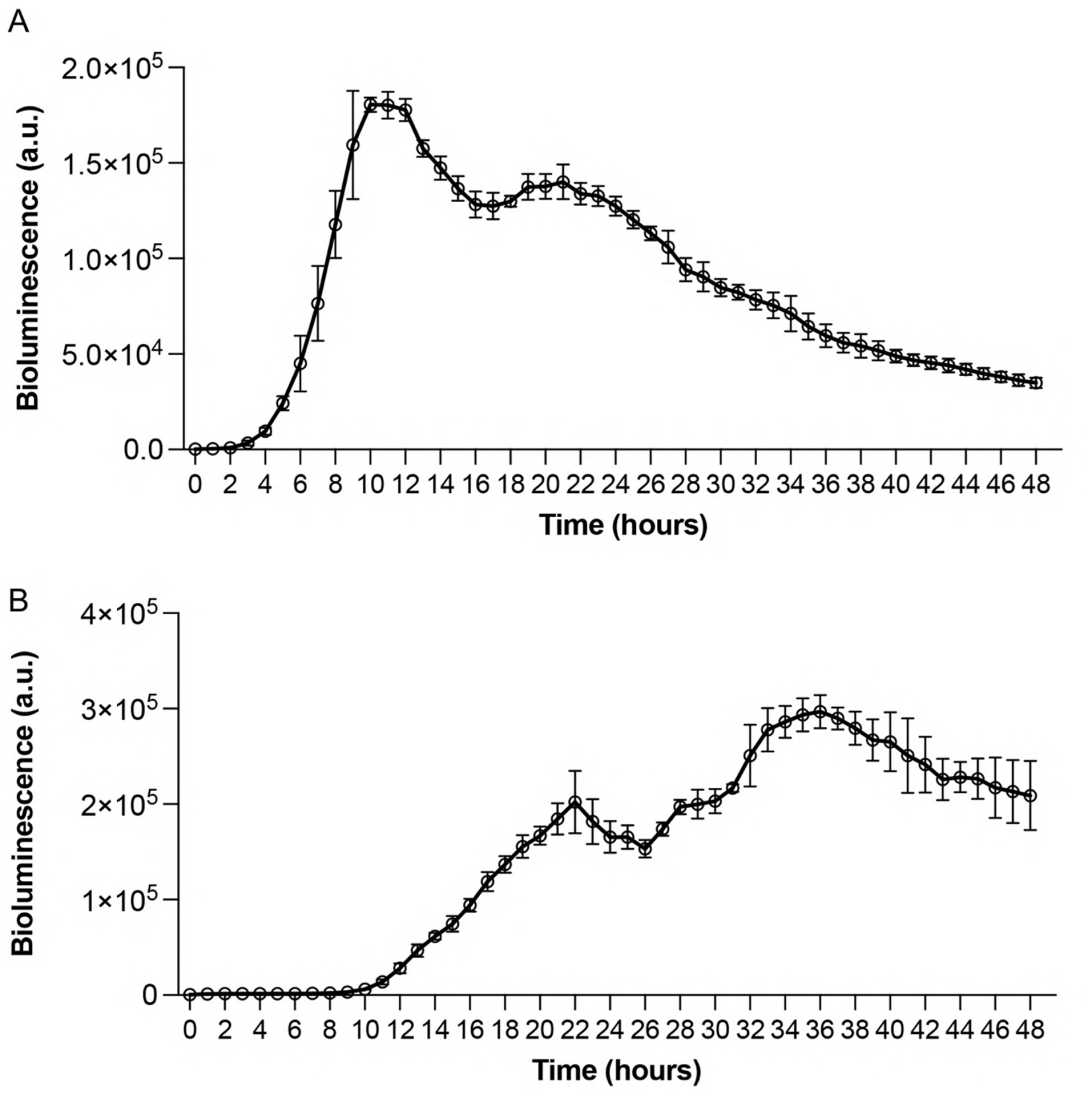
Time-dependent bioluminescence profiles of *Pseudomonas aeruginosa* Xen 41 and *Staphylococcus aureus* 229 during exponential growth phase. Standardized bacterial suspensions (5 × 10^5^ CFU/mL) were monitored for light emission under controlled conditions. **(A)** Real-time bioluminescence measurements of *Pseudomonas aeruginosa* Xen 41 demonstrating growth-dependent light emission patterns. **(B)** Bioluminescence profile of *Staphylococcus aureus* 229 showing strain-specific emission kinetics. Measurements were recorded hourly for 48 h at 32 °C under aerobic conditions. Data points represent mean bioluminescence in arbitrary units (a.u.) ± SEM from three independent experiments (*n = 3*). The continuous monitoring reveals distinct patterns of metabolic activity and growth phases for each bacterial strain.

To evaluate the usefulness of bioluminescence as a real-time indicator of bacterial viability and metabolic activity, we performed thorough kinetic analyses using white 96-well immunoplates. This approach was designed to overcome the limitations of traditional growth measurements by enabling continuous, real-time monitoring of bacterial responses without requiring physical sampling or culture disruption. We utilized standard MIC assay culturing conditions in which bacterial suspensions (5 × 10^5^ CFU/mL) were incubated at 32 °C. Results showed distinct strain-specific patterns with *P. aeruginosa* Xen 41 displaying constant luminescence for the first 2 h, followed by exponential increase at hour 3, reaching maximum luminescence at hour 10, with subsequent gradual decline after hour 11 (Figure 2A). In contrast, *S. aureus* 229 exhibited a longer adaptation period with a 9-h lag phase, followed by exponential increase until reaching peak luminescence at hour 36, and declining after hour 37 (Figure 2B). The biphasic bioluminescence reflects metabolic transitions during growth, similar to diauxic patterns observed in bacterial systems (Sato and Sasaki, 2008; Chu and Barnes, 2016). The first peak (~24 h) corresponds to primary carbon source utilization, while the second (~36 h) arises from adaptation to secondary substrates. These distinct bioluminescence profiles provided vital baseline data for subsequent antimicrobial efficacy studies and demonstrated the sensitivity of the bioluminescence assay in detecting metabolic changes. While optical density detects total cell mass - including dormant or non-viable cells - bioluminescence specifically reflects active metabolic processes, which explains the initial absence of luminescence at lower bacterial densities and highlights the assay’s sensitivity to metabolic changes during bacterial growth.

### 3.3 Kinetic studies of antimicrobial effects on *Pseudomonas aeruginosa* Xen 41

**Figure 3:**
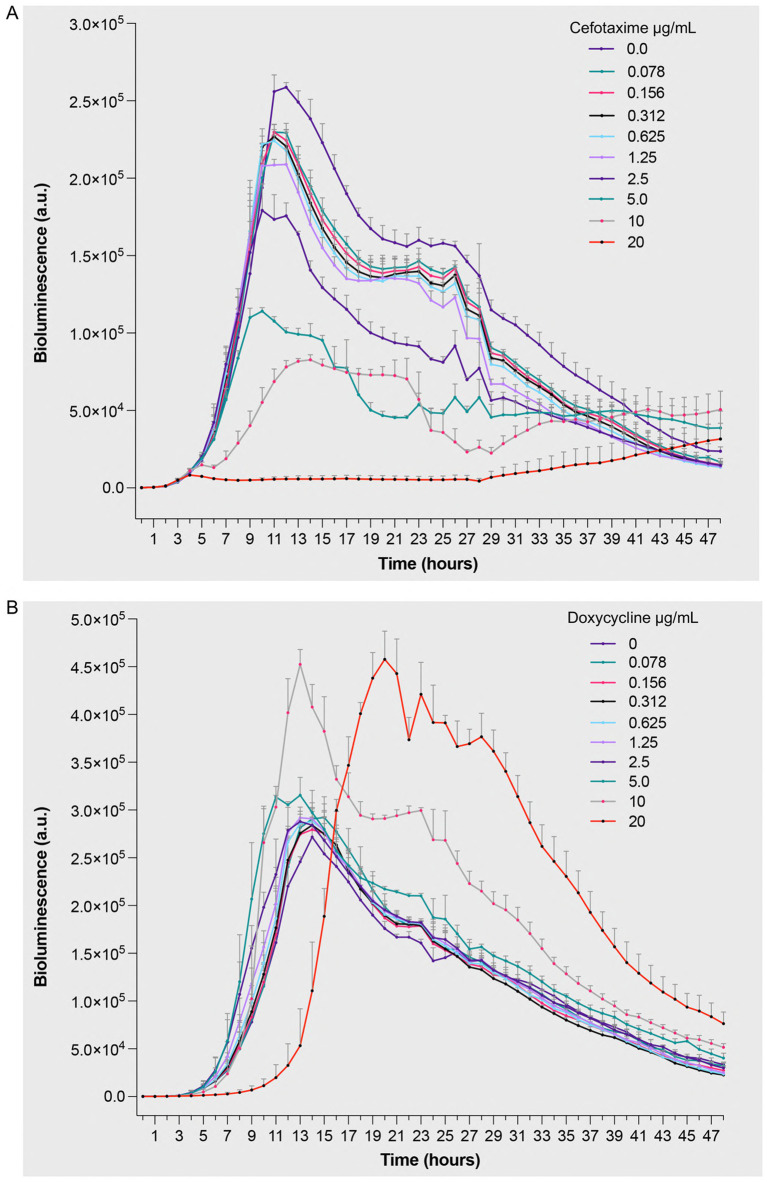
Bioluminescent response of *Pseudomonas aeruginosa* Xen 41 to antibiotic treatment. Bacterial suspensions (5 × 10^5^ CFU/mL) were exposed to increasing concentrations (0–20 μg/mL) of **(A)** cefotaxime, a *β*-lactam antibiotic targeting cell wall synthesis, and **(B)** doxycycline, a protein synthesis inhibitor. Bioluminescence intensity was monitored hourly over 48 h of incubation at 32 °C under aerobic conditions. The measured values represent real-time quantification of bacterial metabolic activity and viability in response to antimicrobial treatment, where decreased bioluminescence indicates reduced metabolic activity or cell death. Data are presented as mean arbitrary units (a.u.) ± SEM from three independent experiments performed (*n = 3*). *p* values were calculated using a two-way ANOVA with Fisher’s LSD test. Full statistical details, including *p*-values for all time points and concentrations, are provided in Supplementary Table 1.

To evaluate real-time antimicrobial efficacy and understand the temporal dynamics of bacterial responses to antibiotics, we performed kinetic studies using bioluminescence assays. This approach was chosen for its ability to provide immediate, non-invasive measurements of bacterial metabolic activity. Standardized bacterial suspensions (5 × 10^5^ CFU/mL) were exposed to increasing concentrations (0–20 μg/mL) of antimicrobial agents, with measurements taken over 48 h. Treatment with cefotaxime induced a dose-dependent reduction in bioluminescent signal compared to untreated controls (Figure 3A and Supplementary Table 1). Higher cefotaxime concentrations reduced bioluminescence starting at 6 h (10 μg/mL), with maximal suppression observed by 24–30 h. The impact became less pronounced after 35 h as bioluminescence in the control group also began to decline. This observation aligns with the drug’s mechanism of action, where cell wall synthesis inhibition leads to decreased metabolic activity and eventual cell death. The direct correlation between increasing cefotaxime concentrations and diminishing bioluminescence demonstrates the assay’s sensitivity in detecting antibiotic-induced metabolic changes. Doxycycline exhibited a distinct effect pattern, characterized by minor dose-dependent extension of the lag phase, consistent with its bacteriostatic activity. At 20 μg/mL, bacterial growth was delayed by 15 h compared to the 6-h lag in untreated samples (Figure 3B and Supplementary Table 1). Higher drug concentrations significantly reduced bioluminescence starting from 17 h, with more pronounced effects observed around 21–23 h. The extended lag phase reflects doxycycline’s protein synthesis inhibition mechanism, where bacterial adaptation to the antibiotic stress requires additional time before growth resumption.

### 3.4 Antimicrobial effects on *Staphylococcus aureus* 229

**Figure 4:**
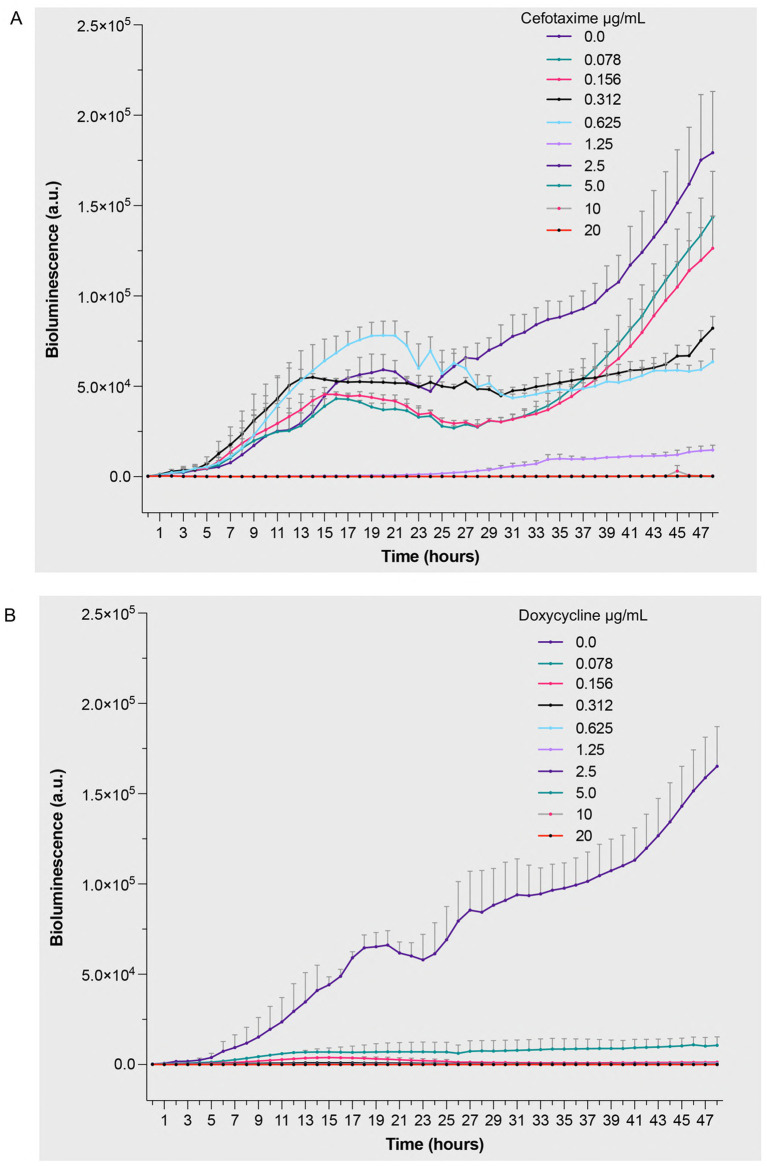
Bioluminescent response of *Staphylococcus aureus* 229 to antibiotic treatment. Bacterial suspensions (5 × 10^5^ CFU/mL) were exposed to increasing concentrations (0–20 μg/mL) of **(A)** cefotaxime, a β-lactam antibiotic targeting cell wall synthesis, and **(B)** doxycycline, a protein synthesis inhibitor. Bioluminescence intensity was monitored hourly over 48 h of incubation at 32 °C under aerobic conditions. The measured values represent real-time quantification of bacterial metabolic activity and viability in response to antimicrobial treatment, where decreased bioluminescence indicates reduced metabolic activity or cell death. Data are presented as mean arbitrary units (a.u.) ± SEM from three independent experiments (*n = 3*). *p* values were calculated using a two-way ANOVA with Fisher’s LSD test. Full statistical details, including *p*-values for all time points and concentrations, are provided in Supplementary Table 1.

To compare antimicrobial efficacy across different bacterial species and validate the broad applicability of the bioluminescence approach, we performed parallel studies with *S. aureus* 229 under identical conditions. Cefotaxime treatment resulted in concentration-dependent reduction of bioluminescent signal (Figure 4A and Supplementary Table 1). Most drug concentrations (1.25–20 μg/mL) significantly reduced bioluminescence starting from 15 h and continued to show effects throughout the 48-h period. The apparent increase of bioluminescence at sub-inhibitory cefotaxime concentrations (≤0.625 μg/mL) up to 25 h aligns with studies showing transient metabolic stress responses at low antibiotic doses (Tenhami et al., 2001), which can temporarily upregulate ATP-dependent pathways. The dose–response relationship provided quantitative evidence of cefotaxime’s bactericidal effects against *S. aureus* 229. Doxycycline showed remarkable efficacy, with significant growth inhibition observed during the incubation period (Figure 4B and Supplementary Table 1). Most drug concentrations significantly reduced bioluminescence starting from 15 h and continued to show effects throughout the 48-h period. The clear dose-dependent response pattern validates the assay’s utility in determining minimum inhibitory concentrations while providing real-time monitoring of bacterial responses to antimicrobial treatment. The absence of a biphasic response and the sustained luminescence observed in antibiotic-free controls likely resulted from gentle shaking during incubation, which enhanced oxygen availability to maintain metabolic activity, contrasting with static conditions that induced localized oxygen depletion. These findings demonstrate the versatility of bioluminescence-based approaches in evaluating antimicrobial efficacy across different bacterial species and antibiotic classes, while providing mechanistic insights through temporal resolution of bacterial responses.

### 3.5 Quantitative assessment of antimicrobial susceptibility using MIC and MBC methods

To validate the bioluminescence findings and establish standardized antimicrobial efficacy parameters, we conducted classical MIC and MBC assays using both antibiotics tested in this study. These well-established methods serve as essential reference points for comparing novel approaches and provide quantitative measures of antimicrobial activity. MIC and MBC assays were performed using standardized bacterial inoculum (5 × 10^5^ CFU/mL) in MH broth, with appropriate controls. Antibiotic concentrations ranging from 0–20 μg/mL were tested in a 96-well plate format. The differential responses of the two bacterial strains to antimicrobial treatment revealed important insights into their susceptibility profiles. *P. aeruginosa* Xen 41 demonstrated considerable resistance to both antibiotics, with cefotaxime showing an MIC of 20 μg/mL and doxycycline exceeding this concentration (Table 1). The high MBC values (>20 μg/mL) for both antibiotics against *P. aeruginosa* Xen 41 align with the delayed growth patterns observed in our bioluminescence studies and suggest primarily bacteriostatic activity at the tested concentrations. These findings reflect *P. aeruginosa’*s known intrinsic resistance mechanisms and highlight the challenges in treating infections caused by this pathogen. In contrast, *S. aureus* 229 exhibited markedly higher susceptibility to both antimicrobial agents (Table 1). The strain showed particularly low MIC values for cefotaxime (0.07 μg/mL) and doxycycline (0.15 μg/mL), with corresponding MBC values of 1.25 μg/mL and 0.3 μg/mL, respectively. The relatively small difference between MIC and MBC values, especially for doxycycline, indicates efficient bactericidal activity against this strain. These quantitative results validate the marked reduction in bioluminescence observed in our earlier experiments, validating the sensitivity and reliability of the bioluminescence-based approach for antimicrobial susceptibility testing.

**Table 1:** Minimum inhibitory concentration (MIC) and minimum bactericidal concentration (MBC) values for antimicrobial agents against bioluminescent bacterial strains.

*Pseudomonas aeruginosa* Xen 41		
	Cefotaxime	Doxycycline hyclate
Minimum inhibitory concentration (MIC)	20 μg/ml	> 20 μg/ml
Minimum bactericidal concentration (MBC)	> 20 μg/ml	> 20 μg/ml

*Staphylococcus aureus* 229		
	Cefotaxime	Doxycycline hyclate
Minimum inhibitory concentration (MIC)	0.15 μg/ml	0.07 μg/ml
Minimum bactericidal concentration (MBC)	1.25 μg/ml	0.3 μg/ml

*Pseudomonas aeruginosa* Xen 41 and *Staphylococcus aureus* 229 bacteria were cultured at standardized inoculum density (5 × 10^5^ CFU/ml) and exposed to increasing concentrations (0–20 μg/ml) of cefotaxime and doxycycline. Measurements were determined after 24 h of incubation at 37 °C under aerobic conditions. Values represent mean determinations from three independent experiments (*n* = 3). MIC values indicate the lowest antibiotic concentration preventing visible bacterial growth, while MBC values represent the minimum concentration required for 99.9% reduction in bacterial viability.

## 4 Discussion

The growing challenge of antibiotic resistance and treatment failures necessitates more rigorous scientific studies and the development of innovative strategies to optimize antimicrobial treatments (Canton and Morosini, 2011). Commonly employed laboratory approaches for *in vitro* antibacterial activity assessment such as disk diffusion, broth or agar dilution assays, and MIC assays have significant limitations. Traditional MIC assays rely on visual inspection of bacterial growth with shorter incubation durations and offer limited real-time insights into antimicrobial effects.

Our findings align with emerging bioluminescence-based approaches demonstrated in various experimental systems. The two-step assay developed by Czieborowski et al. for evaluating antibacterial coatings highlights the technique’s versatility in distinguishing surface effects from leaching mechanisms (Czieborowski et al., 2022), while our methodology extends these principles to direct antimicrobial efficacy testing against clinically relevant pathogens.

Although optical density measurements provide real-time data, they cannot differentiate between metabolically active and dormant bacteria, which significantly limits their utility in antimicrobial efficacy studies (Suominen et al., 2020). This limitation has encouraged the development of alternative approaches, particularly bioluminescence-based methods. Viability assessments using luminescent bacteria or those engineered with reporter genes such as bacterial luciferase offer valuable insights due to the direct correlation between light production and metabolic activity (Hassan et al., 2016).

The rapid antibiotic testing system developed by Tenhami et al. which uses inducible firefly luciferase in *S. aureus* achieved results within 1–4 h (Tenhami et al., 2001), while our extended 48-h monitoring with constitutive lux systems captured delayed resistance patterns undetectable in shorter assays. This advancement in temporal resolution addresses a key limitation in assessing antibiotics like doxycycline that exhibit time-dependent pharmacodynamics.

In this study, we followed antimicrobial kinetics in real time using both luminometric and photometric methods. To ensure optimal growth conditions and achieve meaningful comparisons, we employed experimental parameters aligned with standard minimum inhibitory concentration (MIC) assays. This approach also enhances the reliability and reproducibility of our results, allowing for more accurate assessments of antimicrobial efficacy across different testing methods. Growth curve studies using optical density provided baseline growth characteristics of *S. aureus* 229 and *P. aeruginosa* Xen 41. While optical density measurements offer immediate estimates of bacterial cell count, they are subject to instrument-specific variations and require careful standardization (Mira et al., 2022). A temperature of 32 °C was selected for the experimental conditions to minimize sample evaporation while maintaining sufficient bacterial growth and metabolic activity (Suominen et al., 2020). This adjustment helps ensure measurement stability throughout the extended 48-h monitoring period without compromising the assessment of antimicrobial effects.

Our methodology shares conceptual ground with Marcelo et al.’s luminescence-based nanoparticle assessment in *E. coli* (Marcelo et al., 2022), particularly in overcoming optical density limitations. While their model focused on nanomaterial testing challenges, we demonstrate precise differentiation of bacteriostatic vs. bactericidal effects through continuous metabolic monitoring. Consistent with the findings of Vojtek et al. (2014), who showed that bioluminescence intensity provides a rapid, dose-dependent, and real-time measurement of antibacterial activity in insect haemolymph, our study confirms that bioluminescence-based assays reliably reflect bacterial viability and the kinetics of antimicrobial action in clinically relevant pathogens.

Distinct patterns of antimicrobial efficacy were observed using the bioluminescence assay. Cefotaxime treatment markedly reduced the bioluminescent signal in *P. aeruginosa* Xen 41, indicating rapid metabolic inhibition compared to untreated controls. Doxycycline induced a specific delayed growth response, with extended lag phases correlating with increasing concentrations (0–20 μg/mL), revealing dose-dependent effects for both antibiotics.

Similar susceptibility patterns were observed for *S. aureus* 229, with both cefotaxime and doxycycline treatments significantly decreasing bioluminescence signals in a dose-dependent manner. These findings align with previous studies showing the utility of bioluminescence-based approaches for real-time monitoring of antimicrobial efficacy (Suominen et al., 2020; Ogunniyi et al., 2018).

Notably, our dual-strain approach using modified lux operons in both Gram-positive and Gram-negative species under identical experimental conditions represents a significant methodological advancement over previous single-organism systems such as Loimaranta et al. (1998) *S. mutans* model, enabling direct comparison of antimicrobial mechanisms across bacterial classifications.

We employed classical MIC and MBC assays to further validate our findings with bioluminescence assays. The association between these traditional methods and our bioluminescence results supports the reliability of the bioluminescence-based approach for antimicrobial susceptibility testing. Our study shows that bioluminescent bacterial strains, coupled with real-time monitoring, provide a robust platform for evaluating antimicrobial efficacy. The maximum antibiotic concentration tested (20 μg/mL) was selected to match clinically relevant levels. This range was sufficient to compare bioluminescence and traditional MIC/MBC results for *S. aureus* and allowed us to assess metabolic responses to treatment in both strains.

Important findings distinguishing our work include: (1) Cross-species comparison of luxCDABE systems under identical conditions, (2) 48-h kinetic correlation with classical MIC/MBC endpoints, and (3) Temporal resolution of differential responses to cell wall vs. protein synthesis inhibitors - advancements not achievable through conventional methods or short-term bioluminescent assays.

Furthermore, successful application of our method to extended 48-h incubation periods offers insights into antimicrobial efficacy, particularly regarding delayed effects or bacterial adaptive responses. Monitoring bacterial populations over extended time periods may reveal nuanced patterns of antimicrobial activity overlooked by shorter assays, enhancing our understanding of drug-pathogen interactions. This approach offers several advantages over traditional methods, including immediate detection of metabolic inhibition, real-time monitoring of bacterial responses, reduced time to results, and non-reliance on visual inspection. Altogether, these features improve the efficiency and reliability of antimicrobial susceptibility testing, making bioluminescence-based methods a promising alternative or complementary method to conventional approaches.

These findings may have potential applications in both basic research and clinical settings, particularly for rapid screening of antimicrobial compounds. Recent advancements in bioluminescent reporter systems, including ATP-based assays (Sever et al., 2024) and NanoLuc-optimized strains (Farkas et al., 2024), have demonstrated the technique’s potential for rapid antimicrobial evaluation. However, most prior studies focused on single-species models or short-term monitoring (<24 h) (Suominen et al., 2020; Ogunniyi et al., 2018), leaving gaps in understanding extended temporal responses across different bacterial species. For instance, while ATP-bioluminescence methods achieve results within 3 h for *Enterobacterales* (Sever et al., 2024), and NanoLuc systems enable sensitive *in vivo* tracking of *S. aureus* (Shang et al., 2023), these approaches lack comparative analysis of metabolic dynamics in clinically relevant pathogens like *P. aeruginosa* and *S. aureus* over prolonged incubation periods. Our study addresses these limitations by integrating dual-species bioluminescent profiling with 48-h real-time monitoring, providing mechanistic insights into antibiotic-induced metabolic changes. Future studies should focus on standardizing bioluminescence-based methods for clinical applications and expanding their use in the evaluation of combination therapies.

## Data Availability

The raw data supporting the conclusions of this article will be made available by the authors, without undue reservation.
