# Supplementary material for: Real-time evaluation of antibacterial efficacy using bioluminescent assays for Pseudomonas aeruginosa and Staphylococcus aureus
Source: Front Microbiol. 2025 Aug 29;16:1569217. doi: 10.3389/fmicb.2025.1569217 (PMC12426007; doi:10.3389/fmicb.2025.1569217)
Supplement: Supplementary file 1 [file Data_Sheet_1.PDF]

Real-Time Evaluation of Antibacterial Efficacy Using Bioluminescent Assays for *Pseudomonas aeruginosa* and *Staphylococcus aureus* (Patil M. et al.)

Supplementary Table 1.

Statistical analysis of bioluminescence changes in *P. aeruginosa* Xen 41 and *S. aureus* 229 following antibiotic treatment over a 48-hour period. Comparisons were made between various concentrations of antibiotic-treated samples and untreated controls. *$P \leq 0.05$, **$P \leq 0.01$, ***$P \leq 0.001$, ****$P \leq 0.0001$. NS, non-significant. *P* values were calculated using a two-way ANOVA test.

**Comparison** (Control Vs various antibiotic concenetrations in µg/mL)	**P values**			
	***P. aeruginosa* + Cefotaxime**	***P. aeruginosa* + Doxycycline**	***S. aureus* + Cefotaxime**	***S. aureus* + Doxycycline**
**0 hour**				
0 vs. 20	ns	ns	ns	ns
0 vs. 10	ns	ns	ns	ns
0 vs. 5	ns	ns	ns	ns
0 vs. 2.5	ns	ns	ns	ns
0 vs. 1.25	ns	ns	ns	ns
0 vs. 0.625	ns	ns	ns	ns
0 vs. 0.3125	ns	ns	ns	ns
0 vs. 0.15625	ns	ns	ns	ns
0 vs. 0.078125	ns	ns	ns	ns
**1 hour**				
0 vs. 20	ns	ns	ns	*
0 vs. 10	ns	ns	ns	*
0 vs. 5	ns	ns	ns	ns
0 vs. 2.5	ns	ns	ns	ns
0 vs. 1.25	ns	ns	ns	ns
0 vs. 0.625	ns	ns	ns	ns
0 vs. 0.3125	ns	ns	ns	ns
0 vs. 0.15625	ns	ns	ns	ns
0 vs. 0.078125	ns	ns	ns	ns
**2 hour**				
0 vs. 20	ns	ns	*	**
0 vs. 10	ns	ns	*	***
0 vs. 5	ns	ns	*	***
0 vs. 2.5	ns	ns	ns	**
0 vs. 1.25	ns	ns	ns	**
0 vs. 0.625	ns	ns	ns	**
0 vs. 0.3125	ns	ns	ns	**
0 vs. 0.15625	ns	ns	ns	**
0 vs. 0.078125	ns	ns	ns	**

3 hour

0 vs. 20	ns	ns	ns	ns
0 vs. 10	ns	ns	ns	ns
0 vs. 5	ns	ns	ns	ns
0 vs. 2.5	ns	ns	ns	ns
0 vs. 1.25	ns	ns	ns	ns
0 vs. 0.625	ns	ns	ns	ns
0 vs. 0.3125	ns	ns	ns	ns
0 vs. 0.15625	ns	ns	ns	ns
0 vs. 0.078125	ns	ns	ns	ns

4 hour

0 vs. 20	ns	ns	ns	ns
0 vs. 10	ns	ns	ns	ns
0 vs. 5	ns	ns	ns	ns
0 vs. 2.5	ns	ns	ns	ns
0 vs. 1.25	ns	ns	ns	ns
0 vs. 0.625	ns	ns	ns	ns
0 vs. 0.3125	ns	ns	ns	ns
0 vs. 0.15625	ns	ns	ns	ns
0 vs. 0.078125	ns	ns	ns	ns

5 hour

0 vs. 20	*	ns	ns	ns
0 vs. 10	ns	ns	ns	ns
0 vs. 5	ns	ns	ns	ns
0 vs. 2.5	ns	ns	ns	ns
0 vs. 1.25	ns	ns	ns	ns
0 vs. 0.625	ns	ns	ns	ns
0 vs. 0.3125	ns	ns	ns	ns
0 vs. 0.15625	ns	ns	ns	ns
0 vs. 0.078125	ns	ns	ns	ns

6 hour

0 vs. 20	ns	ns	ns	ns
0 vs. 10	ns	ns	ns	ns
0 vs. 5	ns	ns	ns	ns
0 vs. 2.5	ns	ns	ns	ns
0 vs. 1.25	ns	ns	ns	ns
0 vs. 0.625	ns	ns	ns	ns
0 vs. 0.3125	ns	ns	ns	ns
0 vs. 0.15625	ns	ns	ns	ns
0 vs. 0.078125	ns	ns	ns	ns

7 hour

0 vs. 20	ns	ns	ns	ns
0 vs. 10	ns	ns	ns	ns
0 vs. 5	ns	ns	ns	ns
0 vs. 2.5	ns	ns	ns	ns
0 vs. 1.25	ns	ns	ns	ns
0 vs. 0.625	ns	ns	ns	ns
0 vs. 0.3125	ns	ns	ns	ns
0 vs. 0.15625	ns	ns	ns	ns
0 vs. 0.078125	ns	ns	ns	ns

8 hour

0 vs. 20	**	ns	ns	ns
0 vs. 10	**	ns	ns	ns
0 vs. 5	ns	ns	ns	ns
0 vs. 2.5	ns	ns	ns	ns
0 vs. 1.25	ns	ns	ns	ns
0 vs. 0.625	ns	ns	ns	ns
0 vs. 0.3125	ns	ns	ns	ns
0 vs. 0.15625	ns	ns	ns	ns
0 vs. 0.078125	ns	ns	ns	ns

9 hour

0 vs. 20	*	ns	ns	ns
0 vs. 10	*	ns	ns	ns
0 vs. 5	ns	ns	ns	ns
0 vs. 2.5	ns	ns	ns	ns
0 vs. 1.25	ns	ns	ns	ns
0 vs. 0.625	ns	ns	ns	ns
0 vs. 0.3125	ns	ns	ns	ns
0 vs. 0.15625	ns	ns	ns	ns
0 vs. 0.078125	ns	ns	ns	ns

10 hour

0 vs. 20	*	*	ns	ns
0 vs. 10	*	*	ns	ns
0 vs. 5	ns	*	ns	ns
0 vs. 2.5	ns	*	ns	ns
0 vs. 1.25	ns	ns	ns	ns
0 vs. 0.625	ns	ns	ns	ns
0 vs. 0.3125	ns	ns	ns	ns

0 vs. 0.15625	ns	ns	ns	ns
0 vs. 0.078125	ns	ns	ns	ns

11 hour

0 vs. 20	**	ns	ns	ns
0 vs. 10	***	*	ns	ns
0 vs. 5	**	ns	ns	ns
0 vs. 2.5	*	ns	ns	ns
0 vs. 1.25	*	ns	ns	ns
0 vs. 0.625	ns	ns	ns	ns
0 vs. 0.3125	ns	ns	ns	ns
0 vs. 0.15625	ns	ns	ns	ns
0 vs. 0.078125	ns	ns	ns	ns

12 hour

0 vs. 20	****	**	ns	ns
0 vs. 10	****	*	ns	ns
0 vs. 5	****	ns	ns	ns
0 vs. 2.5	**	ns	ns	ns
0 vs. 1.25	*	ns	ns	ns
0 vs. 0.625	ns	ns	ns	ns
0 vs. 0.3125	*	ns	ns	ns
0 vs. 0.15625	*	ns	ns	ns
0 vs. 0.078125	*	ns	ns	ns

13 hour

0 vs. 20	***	*	ns	ns
0 vs. 10	**	**	ns	ns
0 vs. 5	***	ns	ns	ns
0 vs. 2.5	**	*	ns	ns
0 vs. 1.25	*	*	ns	ns
0 vs. 0.625	*	*	ns	ns
0 vs. 0.3125	*	ns	ns	ns
0 vs. 0.15625	ns	ns	ns	ns
0 vs. 0.078125	*	**	ns	ns

14 hour

0 vs. 20	**	ns	ns	ns
0 vs. 10	**	*	ns	ns
0 vs. 5	**	ns	ns	ns
0 vs. 2.5	**	ns	ns	ns
0 vs. 1.25	*	ns	ns	ns

0 vs. 0.625	*	ns	ns	ns
0 vs. 0.3125	*	ns	ns	ns
0 vs. 0.15625	*	ns	ns	ns
0 vs. 0.078125	ns	ns	ns	ns
**15 hour**				
0 vs. 20	**	ns	*	**
0 vs. 10	**	ns	*	**
0 vs. 5	**	ns	*	**
0 vs. 2.5	**	ns	*	**
0 vs. 1.25	*	ns	*	**
0 vs. 0.625	*	ns	ns	**
0 vs. 0.3125	*	ns	ns	**
0 vs. 0.15625	*	ns	ns	*
0 vs. 0.078125	*	ns	ns	**
**16 hour**				
0 vs. 20	**	ns	**	**
0 vs. 10	**	*	**	**
0 vs. 5	**	ns	**	**
0 vs. 2.5	**	ns	**	**
0 vs. 1.25	**	ns	**	**
0 vs. 0.625	*	ns	ns	**
0 vs. 0.3125	*	ns	ns	**
0 vs. 0.15625	*	ns	ns	**
0 vs. 0.078125	*	ns	ns	**
**17 hour**				
0 vs. 20	***	*	**	**
0 vs. 10	***	*	**	**
0 vs. 5	*	ns	**	**
0 vs. 2.5	**	ns	**	**
0 vs. 1.25	**	ns	**	**
0 vs. 0.625	**	ns	ns	**
0 vs. 0.3125	**	ns	ns	**
0 vs. 0.15625	**	ns	ns	**
0 vs. 0.078125	*	ns	ns	***
**18 hour**				
0 vs. 20	****	***	*	*
0 vs. 10	****	*	*	*
0 vs. 5	**	ns	*	*

0 vs. 2.5	**	ns	*	*
0 vs. 1.25	*	ns	*	*
0 vs. 0.625	*	ns	ns	*
0 vs. 0.3125	*	ns	ns	*
0 vs. 0.15625	**	ns	ns	*
0 vs. 0.078125	*	ns	ns	**

<u>19 hour</u>

0 vs. 20	***	**	*	*
0 vs. 10	**	**	*	*
0 vs. 5	***	ns	*	*
0 vs. 2.5	**	ns	*	*
0 vs. 1.25	ns	ns	*	*
0 vs. 0.625	ns	ns	ns	*
0 vs. 0.3125	ns	ns	ns	*
0 vs. 0.15625	ns	ns	ns	*
0 vs. 0.078125	ns	ns	ns	**

<u>20 hour</u>

0 vs. 20	**	**	*	*
0 vs. 10	**	**	*	*
0 vs. 5	**	*	*	*
0 vs. 2.5	**	ns	*	*
0 vs. 1.25	ns	ns	*	*
0 vs. 0.625	ns	ns	ns	*
0 vs. 0.3125	ns	ns	ns	*
0 vs. 0.15625	ns	ns	ns	*
0 vs. 0.078125	ns	ns	ns	**

<u>21 hour</u>

0 vs. 20	**	*	*	**
0 vs. 10	**	***	*	**
0 vs. 5	**	***	*	**
0 vs. 2.5	**	**	*	**
0 vs. 1.25	ns	*	*	**
0 vs. 0.625	ns	*	ns	**
0 vs. 0.3125	ns	*	ns	**
0 vs. 0.15625	ns	ns	ns	**
0 vs. 0.078125	ns	ns	ns	**

<u>22 hour</u>

0 vs. 20	**	**	**	*

0 vs. 10	**	***	**	*
0 vs. 5	**	**	**	*
0 vs. 2.5	**	ns	**	*
0 vs. 1.25	ns	ns	**	*
0 vs. 0.625	ns	ns	ns	*
0 vs. 0.3125	ns	ns	ns	*
0 vs. 0.15625	ns	ns	ns	*
0 vs. 0.078125	ns	ns	*	**
**23 hour**				
0 vs. 20	**	*	*	ns
0 vs. 10	*	****	*	ns
0 vs. 5	***	**	*	ns
0 vs. 2.5	**	*	*	ns
0 vs. 1.25	ns	*	*	ns
0 vs. 0.625	ns	*	ns	ns
0 vs. 0.3125	ns	*	ns	ns
0 vs. 0.15625	ns	*	ns	ns
0 vs. 0.078125	ns	*	ns	ns
**24 hour**				
0 vs. 20	***	*	*	ns
0 vs. 10	**	*	*	ns
0 vs. 5	***	ns	*	ns
0 vs. 2.5	**	ns	*	ns
0 vs. 1.25	*	ns	*	ns
0 vs. 0.625	*	ns	ns	ns
0 vs. 0.3125	*	ns	ns	ns
0 vs. 0.15625	ns	ns	ns	ns
0 vs. 0.078125	ns	ns	ns	ns
**25 hour**				
0 vs. 20	****	****	*	ns
0 vs. 10	*	*	*	ns
0 vs. 5	****	ns	*	ns
0 vs. 2.5	***	ns	*	ns
0 vs. 1.25	*	ns	*	ns
0 vs. 0.625	*	ns	ns	ns
0 vs. 0.3125	*	ns	ns	ns
0 vs. 0.15625	ns	ns	ns	ns
0 vs. 0.078125	*	ns	ns	ns

26 hour

0 vs. 20	****	*	*	ns
0 vs. 10	**	*	*	ns
0 vs. 5	**	ns	*	ns
0 vs. 2.5	*	ns	*	ns
0 vs. 1.25	**	ns	*	ns
0 vs. 0.625	*	ns	ns	ns
0 vs. 0.3125	*	ns	ns	ns
0 vs. 0.15625	*	ns	*	ns
0 vs. 0.078125	ns	ns	*	ns

27 hour

0 vs. 20	****	**	**	ns
0 vs. 10	***	*	**	ns
0 vs. 5	****	ns	**	ns
0 vs. 2.5	**	ns	**	ns
0 vs. 1.25	ns	ns	**	ns
0 vs. 0.625	ns	ns	ns	ns
0 vs. 0.3125	ns	ns	ns	ns
0 vs. 0.15625	ns	ns	*	ns
0 vs. 0.078125	ns	ns	*	ns

28 hour

0 vs. 20	*	**	*	ns
0 vs. 10	*	**	*	ns
0 vs. 5	*	ns	*	ns
0 vs. 2.5	ns	ns	*	ns
0 vs. 1.25	ns	ns	*	ns
0 vs. 0.625	ns	ns	ns	ns
0 vs. 0.3125	ns	ns	ns	ns
0 vs. 0.15625	ns	ns	*	ns
0 vs. 0.078125	ns	ns	*	ns

29 hour

0 vs. 20	***	**	**	*
0 vs. 10	***	*	**	*
0 vs. 5	**	ns	**	*
0 vs. 2.5	**	ns	**	*
0 vs. 1.25	**	ns	**	*
0 vs. 0.625	*	ns	ns	*
0 vs. 0.3125	*	ns	ns	*
0 vs. 0.15625	*	ns	*	ns

0 vs. 0.078125	*	ns	*	*

30 hour

0 vs. 20	***	**	*	ns
0 vs. 10	***	*	*	ns
0 vs. 5	*	ns	*	ns
0 vs. 2.5	***	ns	*	ns
0 vs. 1.25	**	ns	*	ns
0 vs. 0.625	**	ns	ns	ns
0 vs. 0.3125	**	ns	ns	ns
0 vs. 0.15625	**	ns	ns	ns
0 vs. 0.078125	**	ns	ns	ns

31 hour

0 vs. 20	***	**	*	*
0 vs. 10	**	ns	*	*
0 vs. 5	**	ns	*	*
0 vs. 2.5	**	ns	*	*
0 vs. 1.25	**	ns	*	*
0 vs. 0.625	*	ns	ns	*
0 vs. 0.3125	*	ns	ns	*
0 vs. 0.15625	*	ns	ns	*
0 vs. 0.078125	*	ns	ns	*

32 hour

0 vs. 20	***	**	*	*
0 vs. 10	**	ns	*	*
0 vs. 5	**	ns	*	*
0 vs. 2.5	**	ns	*	*
0 vs. 1.25	*	ns	*	*
0 vs. 0.625	*	ns	ns	*
0 vs. 0.3125	*	ns	ns	*
0 vs. 0.15625	*	ns	*	*
0 vs. 0.078125	ns	ns	*	*

33 hour

0 vs. 20	**	**	*	*
0 vs. 10	*	ns	*	*
0 vs. 5	*	ns	*	*
0 vs. 2.5	*	ns	*	*
0 vs. 1.25	*	ns	*	*
0 vs. 0.625	ns	ns	*	*

0 vs. 0.3125	ns	ns	*	*
0 vs. 0.15625	ns	ns	*	*
0 vs. 0.078125	ns	ns	*	*
**34 hour**				
0 vs. 20	**	*	*	*
0 vs. 10	*	ns	*	*
0 vs. 5	*	ns	*	*
0 vs. 2.5	*	ns	*	*
0 vs. 1.25	*	ns	*	*
0 vs. 0.625	ns	ns	ns	*
0 vs. 0.3125	ns	ns	ns	*
0 vs. 0.15625	ns	ns	*	*
0 vs. 0.078125	ns	ns	*	*
**35 hour**				
0 vs. 20	**	*	**	*
0 vs. 10	*	ns	**	*
0 vs. 5	*	ns	**	*
0 vs. 2.5	*	ns	**	*
0 vs. 1.25	*	ns	**	*
0 vs. 0.625	ns	ns	*	*
0 vs. 0.3125	ns	ns	*	*
0 vs. 0.15625	ns	ns	*	*
0 vs. 0.078125	ns	ns	*	*
**36 hour**				
0 vs. 20	*	*	*	*
0 vs. 10	ns	ns	*	*
0 vs. 5	ns	ns	*	*
0 vs. 2.5	*	ns	*	*
0 vs. 1.25	ns	ns	*	*
0 vs. 0.625	ns	ns	*	*
0 vs. 0.3125	ns	ns	*	*
0 vs. 0.15625	ns	ns	*	*
0 vs. 0.078125	ns	ns	*	*
**37 hour**				
0 vs. 20	*	*	*	*
0 vs. 10	ns	ns	*	*
0 vs. 5	ns	ns	*	*
0 vs. 2.5	ns	ns	*	*

0 vs. 1.25	ns	ns	*	*
0 vs. 0.625	ns	ns	*	*
0 vs. 0.3125	ns	ns	*	*
0 vs. 0.15625	ns	ns	*	*
0 vs. 0.078125	ns	ns	ns	*
**38 hour**				
0 vs. 20	*	*	*	*
0 vs. 10	ns	ns	*	*
0 vs. 5	ns	ns	*	*
0 vs. 2.5	ns	ns	*	*
0 vs. 1.25	ns	ns	*	*
0 vs. 0.625	ns	ns	*	*
0 vs. 0.3125	ns	ns	*	*
0 vs. 0.15625	ns	ns	*	*
0 vs. 0.078125	ns	ns	ns	*
**39 hour**				
0 vs. 20	ns	*	*	*
0 vs. 10	ns	ns	*	*
0 vs. 5	ns	ns	*	*
0 vs. 2.5	ns	ns	*	*
0 vs. 1.25	ns	ns	*	*
0 vs. 0.625	ns	ns	ns	*
0 vs. 0.3125	ns	ns	ns	*
0 vs. 0.15625	ns	ns	ns	*
0 vs. 0.078125	ns	ns	ns	*
**40 hour**				
0 vs. 20	ns	ns	*	*
0 vs. 10	ns	ns	*	*
0 vs. 5	ns	ns	*	*
0 vs. 2.5	ns	ns	*	*
0 vs. 1.25	ns	ns	*	*
0 vs. 0.625	ns	ns	ns	*
0 vs. 0.3125	ns	ns	ns	*
0 vs. 0.15625	ns	ns	ns	*
0 vs. 0.078125	ns	ns	ns	*
**41 hour**				
0 vs. 20	ns	ns	*	*
0 vs. 10	ns	ns	*	*

0 vs. 5	ns	ns	*	*
0 vs. 2.5	ns	ns	*	*
0 vs. 1.25	ns	ns	*	*
0 vs. 0.625	ns	ns	ns	*
0 vs. 0.3125	ns	ns	ns	*
0 vs. 0.15625	ns	ns	ns	*
0 vs. 0.078125	ns	ns	ns	*

42 hour

0 vs. 20	ns	ns	*	*
0 vs. 10	ns	*	*	*
0 vs. 5	ns	ns	*	*
0 vs. 2.5	ns	ns	*	*
0 vs. 1.25	ns	ns	*	*
0 vs. 0.625	ns	ns	ns	*
0 vs. 0.3125	ns	ns	ns	*
0 vs. 0.15625	ns	ns	ns	*
0 vs. 0.078125	ns	ns	ns	*

43 hour

0 vs. 20	ns	ns	*	*
0 vs. 10	ns	*	*	*
0 vs. 5	ns	ns	*	*
0 vs. 2.5	ns	ns	*	*
0 vs. 1.25	ns	ns	*	*
0 vs. 0.625	ns	ns	ns	*
0 vs. 0.3125	ns	ns	ns	*
0 vs. 0.15625	ns	ns	ns	*
0 vs. 0.078125	ns	ns	ns	*

44 hour

0 vs. 20	ns	ns	*	*
0 vs. 10	ns	*	*	*
0 vs. 5	ns	ns	*	*
0 vs. 2.5	ns	ns	*	*
0 vs. 1.25	ns	ns	*	*
0 vs. 0.625	ns	ns	ns	*
0 vs. 0.3125	ns	ns	ns	*
0 vs. 0.15625	ns	ns	ns	*
0 vs. 0.078125	ns	ns	ns	*

45 hour

0 vs. 20	ns	ns	*	*
0 vs. 10	ns	*	*	*
0 vs. 5	ns	*	*	*
0 vs. 2.5	ns	ns	*	*
0 vs. 1.25	ns	ns	*	*
0 vs. 0.625	ns	ns	ns	*
0 vs. 0.3125	ns	ns	ns	*
0 vs. 0.15625	ns	ns	ns	*
0 vs. 0.078125	ns	ns	ns	*

<u>46 hour</u>

0 vs. 20	ns	ns	*	*
0 vs. 10	ns	*	*	*
0 vs. 5	ns	ns	*	*
0 vs. 2.5	ns	ns	*	*
0 vs. 1.25	ns	ns	*	*
0 vs. 0.625	ns	ns	ns	*
0 vs. 0.3125	ns	ns	ns	*
0 vs. 0.15625	ns	ns	ns	*
0 vs. 0.078125	ns	ns	ns	*

<u>47 hour</u>

0 vs. 20	ns	ns	*	*
0 vs. 10	ns	*	*	*
0 vs. 5	ns	ns	*	*
0 vs. 2.5	ns	ns	*	*
0 vs. 1.25	ns	ns	*	*
0 vs. 0.625	ns	ns	ns	*
0 vs. 0.3125	ns	ns	ns	*
0 vs. 0.15625	ns	ns	ns	*
0 vs. 0.078125	ns	ns	ns	*

<u>48 hour</u>

0 vs. 20	ns	ns	*	*
0 vs. 10	ns	*	*	*
0 vs. 5	ns	ns	*	*
0 vs. 2.5	ns	ns	*	*
0 vs. 1.25	ns	ns	*	*
0 vs. 0.625	*	ns	ns	*
0 vs. 0.3125	ns	ns	ns	*
0 vs. 0.15625	ns	ns	ns	*
0 vs. 0.078125	ns	ns	ns	*
